# Unusual evolution of leiomyosarcoma of the rectum: a case report and review of the literature

**DOI:** 10.1186/s13256-016-1047-8

**Published:** 2016-09-15

**Authors:** N. Sahli, M. Khmou, J. Khalil, S. Elmajjaoui, B. El Khannoussi, T. Kebdani, H. Elkacemi, N. Benjaafar

**Affiliations:** 1 Department of Radiotherapy, National Institute of Oncology, University Mohammed V, Rabat, Morocco; 2 Department of Pathology, National Institute of Oncology, University Mohammed V, Rabat, Morocco

**Keywords:** Leiomyosarcoma, Rectum, Treatment, Evolution

## Abstract

**Background:**

Leiomyosarcoma of the rectum is a rare entity that comprises less than 0.1 % of all rectal malignancies. Given the uncommon nature of this tumor and the controversy about its treatment we report one case and review the literature in an attempt to report a particular evolution and to discuss the most appropriate treatment.

**Case presentation:**

This case report describes the presentation of leiomyosarcoma of the rectum. A 30-year-old man from the north of Morocco presented with rectorrhagia and constipation. On physical examination we found a mass in his rectum approximately 6 cm from his anal margin. Pelvic magnetic resonance imaging showed a rectal mass with a parietal attachment that invaded the fascia and his perirectal tissue. Before any treatment he defecated spontaneously the tumor. On histopathological examination a diagnosis of leiomyosarcoma was made. An anterior resection of his rectum was performed with adjuvant radiotherapy at a dose of 50 Gy. After 1 year of surveillance, he has not presented any clinical symptoms and pelvic magnetic resonance imaging was normal.

Unfortunately, histological analysis of a superficial biopsy of a rectal leiomyosarcoma may not be reflective of the entire tumor mass, and a diagnosis is based essentially on postoperative pathological examination. The optimal treatment modality in patients with rectal leiomyosarcomas is controversial. Prognosis is also poor; tumor size, histological grade, mitotic index, and local staging are the most known prognosis factors.

**Conclusion:**

The prognosis of rectal leiomyosarcoma is poor; more investigations are necessary to understand the progression of these tumors and to define an optimal treatment modality.

## Background

Leiomyosarcoma of the rectum is a rare malignant tumor representing less than 0.1 % of all rectal malignancies [1]. People can have this tumor at any age but it has a particular predilection for the fifth and sixth decades of life and it occurs more frequently in men. Given this uncommon localization of leiomyosarcoma, our case report adds another case to the literature and we will review available data in the literature.

## Case presentation

We describe the case of a 30-year-old man from the north of Morocco with no medical or surgical history and no family history of rectal disease. His dietary habits were characterized by a well-balanced consumption of meat and vegetables. He presented with a 1-year history of rectorrhagia and constipation.

**Fig. 1:**
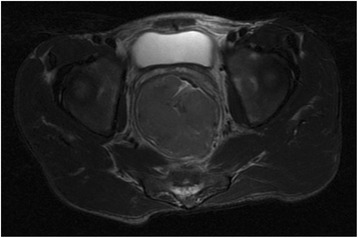
Image showing axial view of a T2-weighted magnetic resonance image of the pelvis, which showed a mass in the rectum

**Fig. 2:**
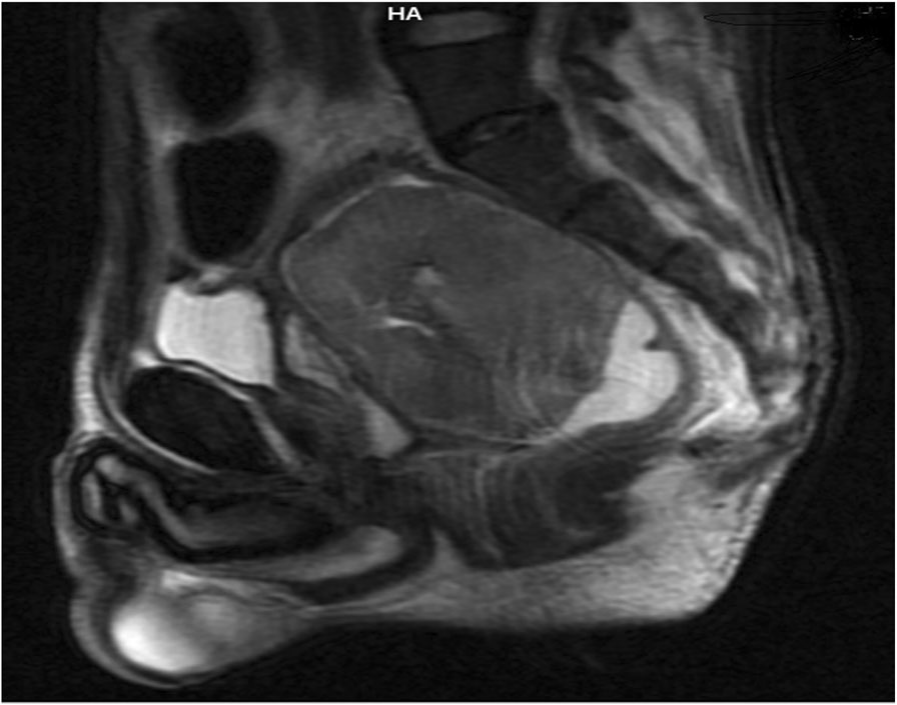
Image showing sagittal view of a T2-weighted magnetic resonance image of the pelvis, which showed a mass in the rectum

A digital rectal examination and rectoscopy revealed a mass in a left posterolateral position in his rectum approximately 6 cm from his anal margin that extended up to 10 cm; the tumor was not mobile. The rest of his physical examination was normal. During rectoscopy, the tumor was biopsied and histological analysis gave no evidence of malignancy. An abdominal and pelvic magnetic resonance imaging revealed an approximately 7.5-cm heterogeneous mass in his rectum. The tumor presented a parietal pedicle with an implantation base of 4 cm and invaded the fascia and the perirectal tissue. There was no evidence of distant metastasis (Figs. 1 and 2).

On biologic examination, his hemoglobin was 10 g/dl and his white blood cells were 7000. His renal function was correct and creatinine clearance was 120 ml/minute. Hepatitis B and C serology was negative. He was hospitalized in our surgical department when he defecated spontaneously the tumor mass.

**Fig. 3:**
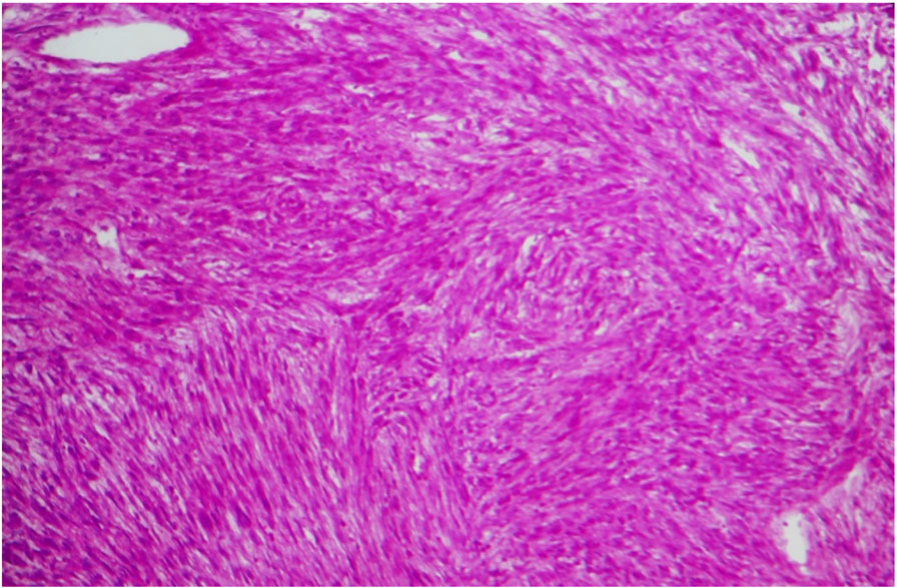
Microscopic examination showing spindle cells proliferation, with nuclei of various sizes and shapes

**Fig. 4:**
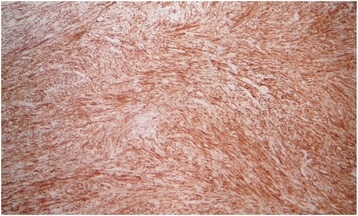
Positive immunostaining for desmin

Macroscopic pathology examination showed a large fleshy lobulated mass measuring 8 × 5 × 3 cm, with foci of hemorrhage. Microscopic examination revealed a cellular tumor arranged in interlacing bundles of spindle-shaped cells, with a marked cellular pleomorphism, a large and hyperchromatic nuclei, and abundant eosinophilic cytoplasm. Mitotic figures (12 per 10 high-power fields) and atypical mitotic figures were also noted. Immunohistochemistry revealed that neoplastic cells expressed desmin, h-caldesmon, and smooth muscle actin, and exhibited negative staining for CD117, CD34, and S-100. Subsequently, a diagnosis of grade II leiomyosarcoma was retained (Figs. 3 and 4).

He had an anterior rectal resection 15 days later; there were no stigma of macroscopic tumor perioperatively. An anatomopathologic examination did not find any residual malignancy.

**Fig. 5:**
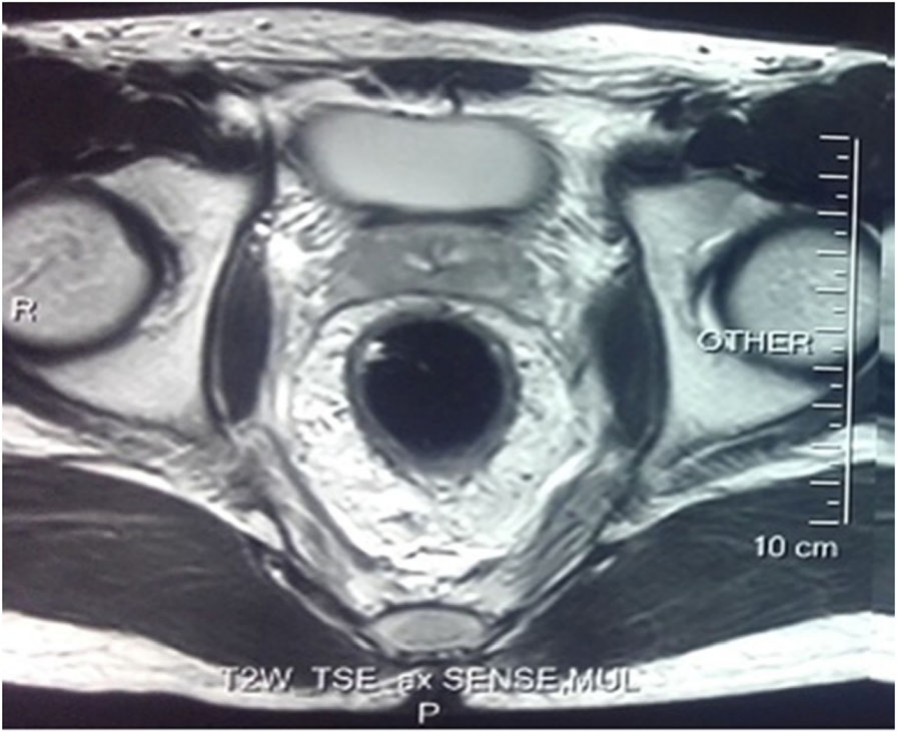
Image showing axial view of a T2-weighted magnetic resonance image of the pelvis, which showed no evidence of recurrence

**Fig. 6:**
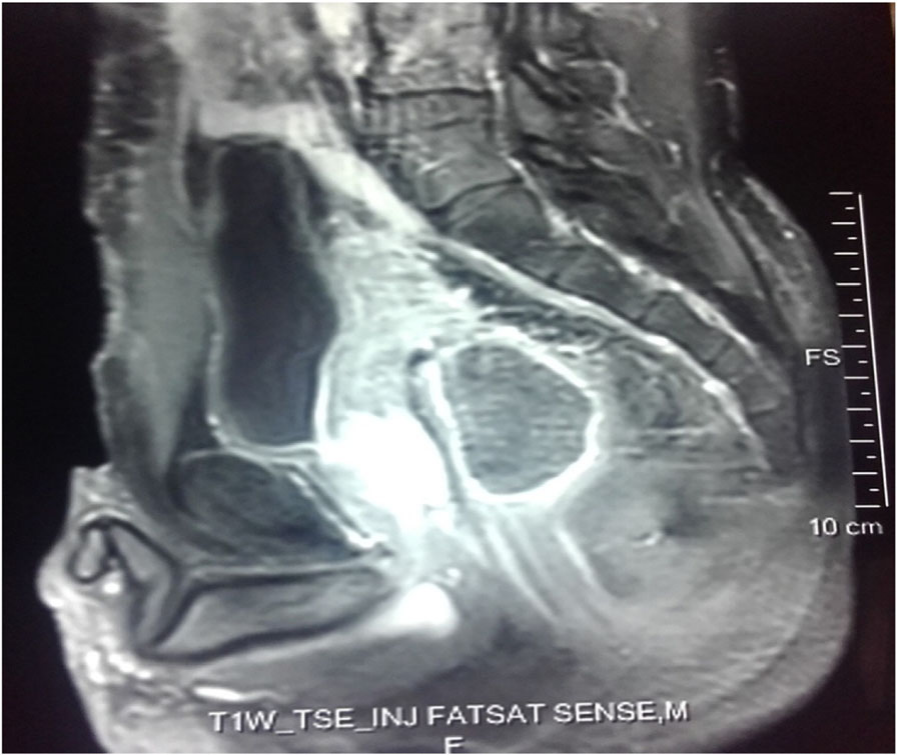
Image showing sagittal view of a T2-weighted magnetic resonance image of the pelvis, which showed no evidence of recurrence

This case was discussed in a multidisciplinary meeting. Because of the initial tumor size and the differentiation degree, postoperative pelvic radiotherapy was indicated for the purpose of improving local control. Three-dimensional radiotherapy of the tumor bed was delivered using three fields (anterior, lateral left, and lateral right), the total prescribed dose was 50 Gy in 2 Gy per fraction, and the treatment duration was 36 days. At the end of radiotherapy, he had follow-up consultation every 3 months. At 1-year post-radiotherapy he is doing well and has no symptoms. Magnetic resonance imaging of his pelvis performed at 6 months post-radiotherapy showed no sign of recurrence (Figs. 5 and 6).

## Discussion

Leiomyosarcomas are malignant mesenchymal tumors arising from smooth muscle cells. Primary rectal leiomyosarcomas are rare and constitute less than 0.1 % of colorectal malignancies [1, 2], and the prognosis is still poor [3]. The differentiation between benign leiomyomas of the rectum and leiomyosarcomas may be difficult because they have identical clinical and radiologic manifestations, and the final diagnosis needs to be confirmed by pathological examination. Unfortunately, histological analysis of superficial biopsy specimens may not be reflective of the entire tumor mass and leiomyosarcomas can be misdiagnosed as benign leiomyomas. Some studies suggest the preoperative histological diagnosis of rectal tumors is adequate in only 29 % of cases [4]. In our case the histological type was not determined even with two biopsies of the rectum; the final diagnostic was retained on the pathological analysis of the expulsed tumor.

On macroscopic examination, leiomyosarcoma is usually large and of a rubbery firm consistency, adherent to the surrounding tissues. Foci of necrosis hemorrhage and cystic degeneration may be seen [5]. On histopathologic examination, typical leiomyosarcoma is characterized by sheets of densely packed spindle cells; neoplastic cells are elongated with abundant fibrillar eosinophilic cytoplasm. Centrally placed, the nucleus is usually blunt-ended or “cigar-shaped.” In some cells, a vacuole is seen at one end of the nucleus. In poorly differentiated tumors the nucleus is more pleomorphic. Multinucleated giant cells may be seen. Necrosis, hemorrhage, and mitotic figures are frequent in these pleomorphic tumors. On immunohistochemical examination, the tumor cells express smooth muscle cell markers: HHF35, desmin, smooth muscle myosin heavy chain, h-caldesmon, and recently transgelin [5, 6].

Leiomyosarcoma must be distinguished from fibromatoses, schwannomas, and gastrointestinal stromal tumor (GIST). In fact, leiomyosarcomas are composed of cells that consistently express smooth cell actin and desmin, but they are negative for CD34 and CD117, these last are positive in the case of GIST [7].

The optimal treatment modality in patients with rectal leiomyosarcomas remains controversial. Radical surgery, such as anterior resection or abdominoperineal resection, is preferred to wide local excision. In fact, radical surgery is associated with a lower recurrence rate than wide local excision. Khalifa *et al*., in a review of 135 cases of rectal leiomyosarcoma, found a local recurrence rate of 67.5 % with local excision, compared to only 19.5 % with abdominoperineal resection, but there were no differences in survival rates between the two surgery modalities [8–10]. In addition, Anderson *et al*. stated that local excisions of rectal leiomyosarcomas were almost always followed by recurrence no matter what grade of malignancy [11]. However, Quan and Berg also believed that wide local excision provided adequate local control for tumors less than 2.0 cm [12].

Because leiomyosarcoma is a relatively radioresistant tumor, and patients with rectal leiomyosarcoma can develop early hematogenous metastases, adjuvant pelvic radiation therapy is generally considered unsuccessful, as for Tjandra *et al*. adjuvant radiation therapy is not effective [13]. In contrast, Minsky *et al*. [14] reported that radiation therapy following conservative surgery can be an alternative to radical surgery with the goals of local control of the disease and rectal sphincter preservation. Adjuvant radiotherapy can be proposed by extension of the management principles of other pelvic tumor types and limb sarcomas, given the absence of randomized clinical trials. Chemotherapy has been generally unsuccessful in treating this tumor. The two most commonly used agents are doxorubicin and dacarbazine [15]. Histopathological characteristics predictive of poor prognosis include high mitotic activity, intratumoral necrosis, and tumor size [16].

In our case, the evolution was unusual compared to other reported cases, with spontaneous expulsion of the entire tumor through the rectum, it may be explained by the necrosis of the tumor pedicle. We did not find cases with such an evolution in the literature, but we found some cases of spontaneous expulsion of ileal lipoma per rectum [17, 18]. As in our case, the mass was connected to the rectum by a pedicle. The gravity of the tumor and the presence of intestinal peristalsis gave rise to ischemia, necrosis, and breakage of the pedicle. Thus, the tumor fell from the rectum.

## Conclusions

Leiomyosarcomas of the rectum are rare tumors with few cases published in the literature. The diagnosis is confirmed by postoperative pathological examination; the overall prognosis is poor with reported survival of 20 to 40 % at 5 years [19]. In view of the rarity of leiomyosarcoma of the rectum, there will probably never be any possibility of a randomized clinical trial to assess the optimal treatment; however, adjuvant radiotherapy can be proposed by extension of the management principles of other pelvic tumor types and limb sarcomas.

## Abbreviation

GIST: Gastrointestinal stromal tumor
